# Automated bone marrow analysis using the CD4000 automated haematology analyser

**DOI:** 10.1155/S1463924600000109

**Published:** 2000

**Authors:** Ryousuke Yamamura, Takahisa Yamane, Masayuki Hino, Kensuke Ohta, Ki-Ryang Koh, Izumi Tsuda, Takayuki Takubo, Noriyuki Tatsumi

**Affiliations:** Department of Clinical Hematology Osaka City University Medical School 1-4-3 Asahimachi Abeno-ku Osaka Japan

## Abstract

At present, bone marrow analysis is performed microscopically, but is time consuming and labour intensive. No automated methods have been successfully applied to classification of bone marrows cells because automated blood cell analysers have been incapable of identifying erythroblasts. The present study was designed to evaluate automated analysis of bone marrow aspirates with the CELL-DYN 4000 (CD4000) haematology analyser, which enables automated determination of erythroblast counts in both the normal mode (haemolytic time; 11.5s) and the resistant RBC mode (34.0s). The percentages of subpopulations including lymphocytes, neutrophils and erythroblasts were obtained with the CD4000, and as a reference, differential counts by microscopic observation of May–Grünwald–Giesa-stained films of bone marrow aspirates were performed (n=98). Significant correlations (P < 0.01) between the results obtained with the two methods were observed for total nucleated cell count and lymphocytes, neutrophils, erythroblasts and myeloid/erythroid (M/E) ratio. However, there were biases in the average percentages of erythroblasts, lymphocytes and M/E ratio obtained using the normal mode with the CD4000 toward values lower than those obtained with the microscopic method. Using the RBC resistant mode with the CD4000, the average percentages of erythroblasts, lymphocytes and M/E ratio approximated those obtained with the microscopic method. In conclusion, the CD4000 in resistant RBC mode is more useful for analysis of bone marrow aspirates than is the normal mode, because the former better approximates the M/E ratio than the latter.


Journal of Automated Methods & Management in Chemistry, Vol. 22, No. 3 (May–June 2000) pp. 89–92

### Automated bone marrow analysis using the CD4000 automated haematology analyser

**Ryousuke Yamamura, Takahisa Yamane[1], Masayuki Hino, Kensuke Ohta, Ki-Ryang Koh, Izumi Tsuda, Takayuki Takubo and Noriyuki Tatsumi**

*Department of Clinical Hematology, Osaka City University Medical School, 1-4-3 Asahimachi, Abeno-ku, Osaka, Japan.*[1] *e-mail: yamane@msic.med.osaka-cu.ac.jp*

*At present, bone marrow analysis is performed microscopically, but is time consuming and labour intensive. No automated methods have been successfully applied to classification of bone marrows cells because automated blood cell analysers have been incapable of identifying erythroblasts. The present study was designed to evaluate automated analysis of bone marrow aspirates with the CELL-DYN 4000 (CD4000) haematology analyser, which enables automated determination of erythroblast counts in both the normal mode (haemolytic time; 11.5 s) and the resistant RBC mode (34.0 s). The percentages of subpopulations including lymphocytes, neutrophils and erythroblasts were obtained with the CD4000, and as a reference, differential counts by microscopic observation of May–Grünwald–Giesa-stained films of bone marrow aspirates were performed ($n = 98$). Significant correlations ($P < 0.01$) between the results obtained with the two methods were observed for total nucleated cell count and lymphocytes, neutrophils, erythroblasts and myeloid/erythroid (M/E) ratio. However, there were biases in the average percentages of erythroblasts, lymphocytes and M/E ratio obtained using the normal mode with the CD4000 toward values lower than those obtained with the microscopic method. Using the RBC resistant mode with the CD4000, the average percentages of erythroblasts, lymphocytes and M/E ratio approximated those obtained with the microscopic method. In conclusion, the CD4000 in resistant RBC mode is more useful for analysis of bone marrow aspirates than is the normal mode, because the former better approximates the M/E ratio than the latter.*

### Introduction

Automated blood cell counters can now accurately perform differential counts of white blood cells in peripheral blood due to technological advances in electronic measurement. Although we have made previous attempts to classify bone marrow cells using blood cell counters, until now it has been impossible to obtain adequate correlations with the results obtained with the microscopic method [1, 2], because blood cell counters have been incapable of identifying erythroblasts and therefore have included them when counting the lymphocyte fraction. The CELL-DYN4000 (CD4000, Abbott Diagnostics, Santa Clara, CA, USA) haematology analyser enables automated determination of erythroblast count [3, 4]. We tested the efficiency of the CD4000 in performing blood cell differentials of bone marrow aspirates.

### Subjects and methods

Ninety-eight patients presenting at the Clinical Hematology Department at Osaka City University Hospital between April and December 1998 were enrolled as subjects in this study. They were patients who had required bone marrow aspiration, but whose samples had revealed no signs of dysplasia in the erythroid or myeloid lines, and who had clean bone marrows free of abnormal cells.

### *Determination by CD4000*

Bone marrow aspirate (0.5 ml) was diluted twofold with 0.5 ml of phosphate-buffered saline, and was then immediately assayed with the CD4000. The CD4000 is a flow cytometer that detects the light scatter emitted by cells when a laser beam is focused on the flow cell. It detects various light scatter data which provide information, e.g. cell size ($0^\circ$, scattered light), intracellular structure ($7^\circ$, scattered light), degree of nuclear segmentation ($90^\circ$ polarized light) and eosinophilic granules ($90^\circ$ depolarized light). With the additional use of fluorescent dyes, it is possible to quantitatively determine DNA and RNA content. These two methods combined enable the CD4000 to detect and count erythroblasts.

### *White blood cell differential*

The major neutrophils which appear in peripheral blood can be differentiated at $0^\circ$ and $90^\circ$ scattered light, whereas eosinophils can be classified by further exposure to $90^\circ$ depolarized light. Neutrophils were primarily differentiated through the $7^\circ$ scattered light which detects the cytoplasmic/nuclear ratio, while lymphocytes and monocytes were differentiated by $90^\circ$ scattered light which particularly reflects the degree of nuclear segmentation.

### *Erythroblast counts*

The CD4000's unique technology is used in the determination of erythroblast counts. In our study, we used two modes for haemolysis with the CD4000, the normal and resistant RBC modes. The time of haemolysis of the resistant RBC mode (34.0 s) is longer than that of the normal mode (11.5 s). During the lysing process non-nucleated erythrocytes are completely destroyed, while normally viable leukocytes are left intact. On the other hand, the lysis process permeates the erythroblast's membrane, but leaves the nucleus intact. This controlled membrane damage allows fluorescent dye, propidium iodide (PI), to bind with DNA in the exposed nuclei. The erythroblasts bound to fluorescent dye can be distinguished from the leukocytes, which remain unstained by PI, through the use of two assay systems (0° scattered light FL3; 90° red fluorescence). The size and scatter characteristics of erythroblasts are usually similar to those of lymphocytes. It is therefore impossible to accurately differentiate lymphocytes from nucleated erythrocytes based on cell size alone. However, by determining the degree of nuclear segmentation along the Y axis (90° polarized light), separation of the nucleated erythrocytes with their simple nuclear composition from the leukocyte line, which has a relatively complex nuclear composition, becomes possible. The leukocytes with DNA staining are assumed to have suffered cell membrane rupture and are therefore judged to be dead. At this point, nucleated erythrocytes and dead cells are excluded from the white cell differential, enabling determination of the nucleated erythrocyte count.

### *Determination of nucleated cell counts in bone marrow aspirate*

The CD4000 yields total white cell counts as well as absolute counts of each type of differentiated leukocyte per given unit of volume. The erythroblast count is generally not expressed as the relative count per 100 leukocytes, which is usually employed clinically, and is instead expressed as the absolute count of nucleated erythrocytes per microlitre. For bone marrow samples, it is imperative that the total nucleated cell count, including erythroblasts, be determined. Therefore, in the CD4000 system, the sum of the WBC and NRBC is considered to be the total nucleated cell count. The erythroblast percentage with the CD4000 was then calculated by dividing the erythroblast count by the sum of the white blood cell and erythroblast counts. The CD4000 was calibrated before the present evaluation, and daily quality control was performed throughout the period of the study according to the manufacturer's manual.

### *Differentiation of cells in bone marrow aspirates by microscopy*

Bone marrow aspirates were obtained from the posterior iliac crest. The volume of each aspirate was 0.5–1.0 ml. For counting, a 1:50 dilution of the marrow with Türk's diluting fluid was performed in a Melangeur blood-diluting pipette for white blood cells. Nucleated cells of these diluted samples were manually determined using a Büker Türk haemocytometer. Smears were prepared on glass slides, and after May–Grünwald–Giemsa staining were observed under a microscope (500 count).

Comparisons were made for the cell counts which could be classified by the CD4000. These included total neutrophils (promyeloblasts, metamyelocytes, myelocytes, band and segmented neutrophils determined by microscopic observation), lymphocytes and erythroblasts. The myeloid/erythroid ratio (M/E ratio) was also calculated.

### *Statistical analysis*

Numerical data are expressed as mean ± SD. Bearmann's test was performed for statistical analysis of data. Differences at risk factors of 0.01 or less were considered significant.

*Table 1. Comparison of subpopulation percentages obtained with the normal mode and the resistant RBC mode with the CD4000 and microscopic observation (mean ± SD).*

| | CD4000 | Manual | Correlation |
|---|---|---|---|
| TNC ($\times 10^9$/l) | | | |
| Resistant | 103.9 ± 8.9 | 114.9 ± 99.9 | 0.93 |
| Normal | 9.2 ± 7.6 | | 0.90 |
| Erythroblasts (%) | | | |
| Resistant | 24.3 ± 8.3 | 29.6 ± 13.4 | 0.68 |
| Normal | 16.1 ± 7.7 | | 0.67 |
| Total neutrophils (%) | | | |
| Resistant | 61.2 ± 13.8 | 50.0 ± 14.8 | 0.66 |
| Normal | 58.4 ± 15.3 | | 0.63 |
| Lymphocytes (%) | | | |
| Resistant | 15.2 ± 7.4 | 12.3 ± 7.1 | 0.82 |
| Normal | 20.9 ± 9.2 | | 0.79 |
| M/E ratio | | | |
| Resistant | 3.3 ± 2.7 | 2.5 ± 2.3 | 0.77 |
| Normal | 5.3 ± 4.0 | | 0.77 |

### Results

For both the normal mode and resistant mode, significant correlations ($P < 0.0001$) between the automated measurements and microscopic ones were obtained for total nucleated cell count and percentages of lymphocytes, immature neutrophils, mature neutrophils, total neutrophils, erythroblasts, and the M/E ratio (table 1, figures 1-5). For the average percentage of the erythroid series, the automated measurements in the normal mode tended to be lower than the microscopic ones. On the other hand, for the average percentage of lymphocytes, the automated measurements in the normal mode tended to be higher than the microscopic ones. For the average percentage of total neutrophils, there were no differences between the automated measurements and the microscopic ones. However, in the resistant mode, for the average percentage of erythroid series and lymphocytes, the automated measurements tended to approximate the microscopic ones, and for the average percentage of total neutrophils, there were no differences between the automated measurements and the microscopic ones. As a result, for the M/E ratio, the automated measurements tended to approximate the microscopic ones in the resistant RBC mode (table 1).

### Discussion

Observation by microscopy is the method traditionally used to classify those blood cells which are found in the bone marrow aspirate. However, blood cell analysers can now perform cell differentials of peripheral blood faster and more accurately than before. These blood cell analysers have been used for the automated analysis of bone marrow cell differentials in addition to the calculation of nucleated cell counts. Although the earlier blood cell analysers were capable of a three-part differential which enabled them to differentiate between lymphocytes, middle-sized cells and neutrophils, the diversity of bone marrow cells made it almost impossible to classify

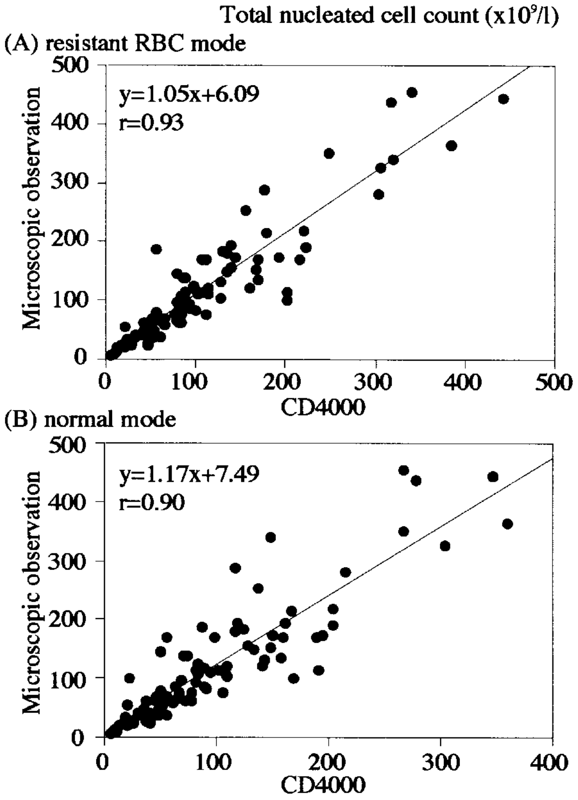

*Figure 1. Correlations for total nucleated cell counts between the CELL-DYN 4000 and the microscopic method (A, resistant RBC mode; B, normal mode).*

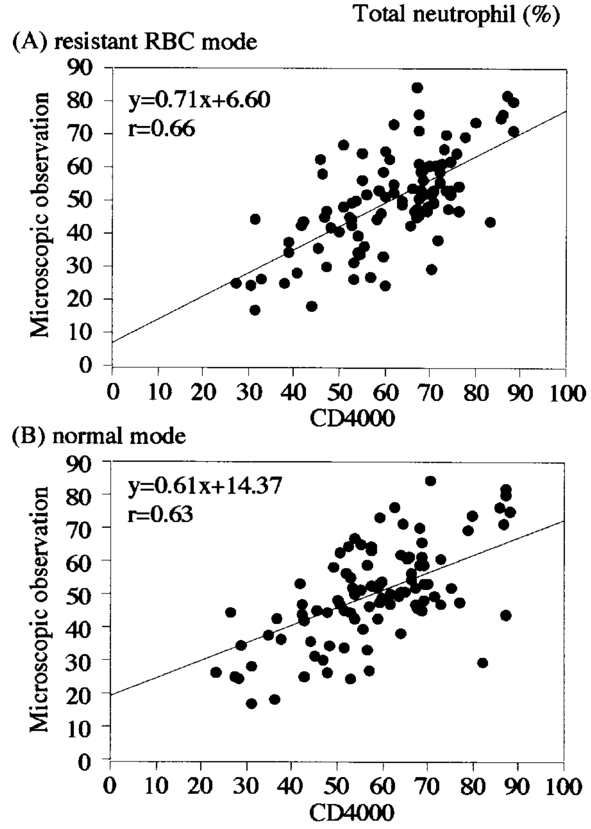

*Figure 3. Correlations for total neutrophil percentages between the CELL-DYN 4000 and the microscopic method (A, resistant RBC mode; B, normal mode).*

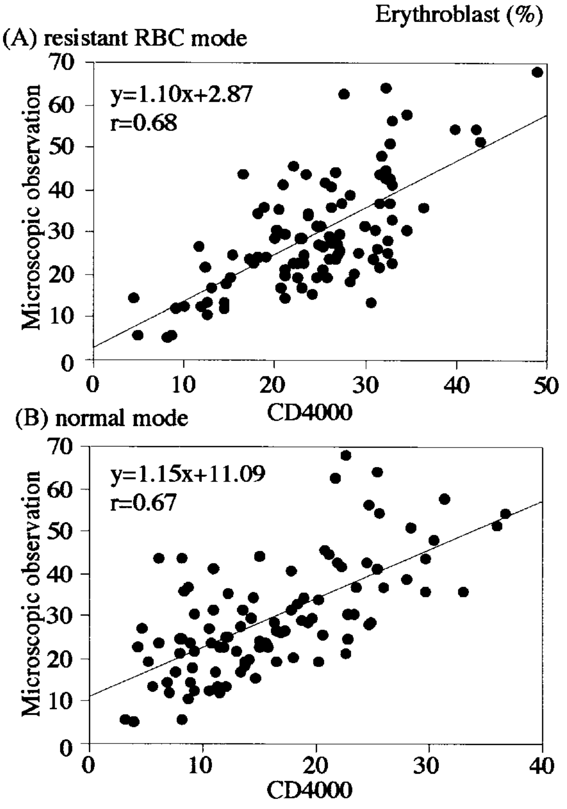

*Figure 2. Correlations for erythroblast percentages between the CELL-DYN 4000 and the microscopic method (A, resistant RBC mode; B, normal mode).*

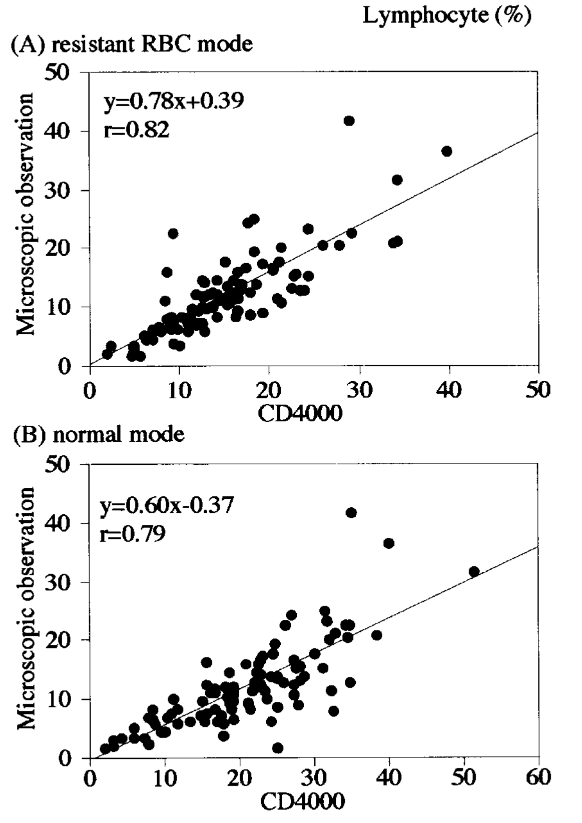

*Figure 4. Correlations for lymphocyte percentages between the CELL-DYN 4000 and the microscopic method (A, resistant RBC mode; B, normal mode).*

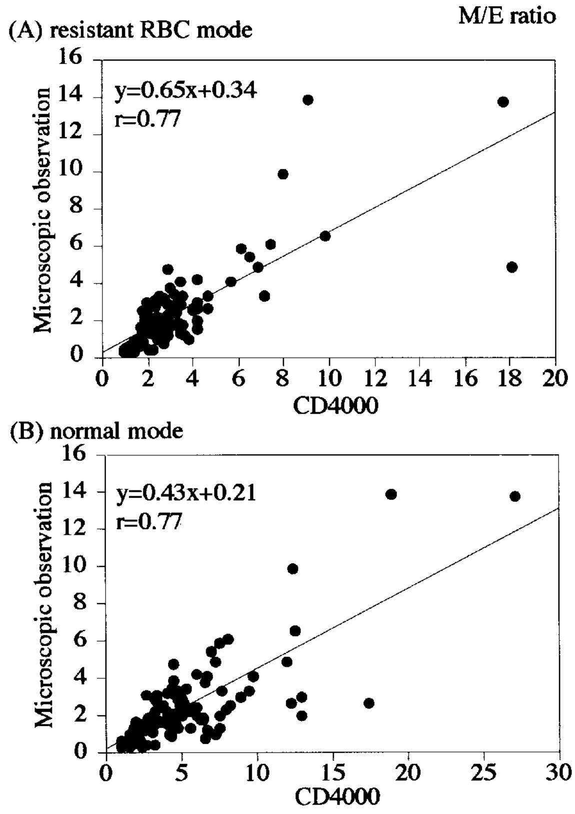

*Figure 5. Correlations for M/E ratio between the CELL-DYN 4000 and the microscopic method (A, resistant RBC mode; B, normal mode).*

them [1, 2]. In particular, erythroblasts were impossible to distinguish from lymphocytes. A five-part differential method was subsequently developed. This method was based upon the fact that lymphocytes and erythroblasts form separate clusters. However, because there was still overlapping between the clusters, it was difficult to clearly separate these two cell groups [5].

The CD4000, which was developed at Abbott Diagnostics, is an automated blood cell analyser which can not only perform automatic five-part peripheral white cell differential counts, it can also detect and count erythroblasts as a separate component [3, 4, 6]. The present study was therefore conducted to compare the conventional microscopic method and CD4000 in differentiation of cells in bone marrow aspirates, in order to determine whether the CD4000 can potentially enable easy, rapid and accurate screening of samples. Previous evaluation of bone marrow aspirates was performed using the resistant RBC mode [7, 8] or the normal mode [9]. In this study, we used both modes simultaneously to estimate total nucleated cell counts and cell differentiations. In this study, significant correlations ($P < 0.0001$) between CD4000 (the normal mode and the resistant RBC mode) and microscopic methods were obtained for total nucleated cell counts, neutrophils, erythroblasts and M/E ratio. However, there were significant differences between the average percentages of erythroblasts, lymphocytes and M/E ratio in the normal mode and those in the resistant RBC mode. Bone marrow aspirates contain a high percentage of erythroblasts compared to peripheral white blood cell components. In the erythroid line, the characteristics of cell membranes differ depending on the stage of cell differentiation, and it is believed that there are some cells which remain resistant to haemolytic agents in the normal mode. Therefore, although the ability of the normal mode with the CD4000 to count erythroblasts is clearly superior to that of other automated blood analysers, the possibility remains that a portion of the erythroblasts remain uncounted with this method and are classified as lymphocytes. We assume that some erythroblasts which were not haemolysed and classified as lymphocytes in the normal mode were completely haemolysed and classified as erythroblasts in the resistant RBC mode. Therefore, the average percentage of the erythroblasts increased and that of the lymphocytes decreased in the resistant RBC mode as compared with in the normal mode. As a result, the average percentages of erythroblasts, lymphocytes and M/E ratio with the automated method approximated those obtained by the microscopic method. The reagents used for the present CD4000 study and the algorithm for differential counts are designed for the analysis of peripheral blood, and there is room for improvement of bone marrow analysis. However, our results have shown the CD4000 to be capable of providing objective findings for percentages of major cell populations, with analysis of at most 10 000 cells in 30–80 s, and that the resistant RBC mode is clinically more useful for analysis of bone marrow aspirates than the normal mode.

### References

1. Tatsumi, N., Yokomatsu, Y., Kojima, K., Setoguchi, K., Im, T. and Tsuda I., *Osaka City Medical Journal*, **34** (1988) 135.
2. Tatsumi, J., Tatsumi, Y. and Tatsumi, N., *American Journal of Clinical Pathology*, **86** (1986) 50.
3. Kim, Y.R., Yee, M., Metha, S., Chupp, V., Kendall, R. and Scott, C.S., *Clinical Laboratory Haematology*, **20** (1998) 21.
4. Sher, G., Vitisallo, B., Schisano, T., Pantalony, D. and Van Hove, L., *Laboratory Hematology*, **3** (1997) 129.
5. Tsuda, I. and Tatsumi, N., *Sysmex Journal*, **13** (1990) 100.
6. Tatsumi, N., Tsuda, I., Shimizu, A., Watanabe, K. and Matsuno, K., *Laboratory Hematology*, **2** (1996) 157.
7. D'Onofrio, G., Zini, G., Tommasi, M., Laurenti, L., Vergini, C. and Van Hove, L., *Laboratory Hematology*, **3** (1997) 146.
8. D'Onofrio, G., Zini, G., Laurenti, L., Hove, L.V., Sica, S. and Leone, G., *Laboratory Hematology*, **4** (1998) 71.
9. Sakamoto, C., Yamane, T., Ohta, K., Hino, M., Tsuda, I. and Tatsumi, N., *Acta Haematologica*, **101** (1999) 130.

